# Giant Subependymoma Developed in a Patient with Aniridia: Analyses of *PAX6* and Tumor-relevant Genes

**DOI:** 10.1111/j.1750-3639.2010.00406.x

**Published:** 2010-11

**Authors:** Motoko Maekawa, Hironori Fujisawa, Yoshimi Iwayama, Akira Tamase, Tomoko Toyota, Noriko Osumi, Takeo Yoshikawa

**Affiliations:** 1 Laboratory for Molecular Psychiatry, RIKEN Brain Science InstituteSaitama; 2 Department of Neurosurgery, Fukui Prefectural HospitalFukui; 3 Department of Neurosurgery, Kanazawa University HospitalIshikawa; 4 Department of Developmental Neurobiology, Tohoku University Graduate School of MedicineSendai; 5 CREST, Japanese Science and Technology AgencyTokyo, Japan

**Keywords:** genomic quantitative PCR, loss of heterozygosity, *PAX6* deletion, *PTEN*, *SOX2*, *TP53*

## Abstract

We observed an unusually large subependymoma in a female patient with congenital aniridia. To analyze the genetic mechanisms of tumorigenesis, we first examined the paired box 6 (*PAX6*) gene using both tumor tissue and peripheral lymphocytes. Tumor suppressor activity has been proposed for *PAX6* in gliomas, in addition to its well-known role in the eye development. Using genomic quantitative PCR and loss of heterozygosity analysis, we identified hemizygous deletions in the 5′-region of *PAX6*. In lymphocytes, the deletion within *PAX6* spanned from between exons 6 and 7 to the 5′-upstream region of the gene, but did not reach the upstream gene, *RNC1*, which is reported to be associated with tumors. The subependymoma had an additional de novo deletion spanning from the intron 4 to intron 6 of *PAX6*, although we could not completely determine whether these two deletions are on the same chromosome or not. We also examined other potentially relevant tumor suppressor genes: *PTEN*, *TP53* and *SOX2*. However, we detected no exonic mutations or deletions in these genes. Collectively, we speculate that the defect in *PAX6* may have contributed to the extremely large size of the subependymoma, due to a loss of tumor suppressor activity in glial cell lineage.

## INTRODUCTION

Subependymoma is a rare, indolent benign tumor of the central nervous system that commonly occurs in middle-aged or elderly men, with an incidence of approximately 0.5% of all intracranial neoplasms (15). The majority of tumors arise in the fourth (60%) and lateral ventricles (30%), and more rarely in the third ventricle, the septum pellucidum and the spinal cord (18). Many subependymomas remain asymptomatic throughout life and are found only incidentally by autopsy or imaging. The pathogenesis of the tumor remains largely unclear.

We observed an extremely rare case of a giant subependymoma in a young female with familial congenital aniridia that spans three generations. Familial congenital aniridia is a hereditary disease transmitted in an autosomal dominant fashion and is caused by genetic defects within the paired box 6 (*PAX6*) gene in about 90% of cases (8). The *PAX6* gene is located at chromosome 11p13, spans 22 kb, consists of 14 exons (including an alternatively spliced exon 5a that encodes 14 amino acids) and encodes a protein of 422 amino acids. PAX6 is a transcription factor that is involved in multiple developmental pathways and is expressed early in the development of the eye, numerous regions of the brain and the pancreas (14). Recently, it has been reported that *PAX6* could be a glioma suppressor gene, based on two main facts: the expression of *PAX6* correlates with astrocytoma grade and survival (24) and PAX6 suppresses the growth of glioblastoma cells *in vitro*(25). It has also been reported that *PAX6* inhibits proliferation of astrocyte progenitors and promotes their maturation in rodents (16). Although the precursor cells of subependymomas have not been conclusively identified, some candidates have been proposed: subependymal glia (1), astrocytes of the subependymal plate, ependymal cells (12) and a mixture of astrocytes and ependymal cells (4).

Since there have been no reports of subependymoma occurring in any hereditary diseases, we set out to perform this study in the hope that analyses of the current case with the complication of eye abnormalities may help determine the mechanisms responsible for the huge growth of the subependymoma.

## MATERIALS AND METHODS

### Patient

**Figure 1:**
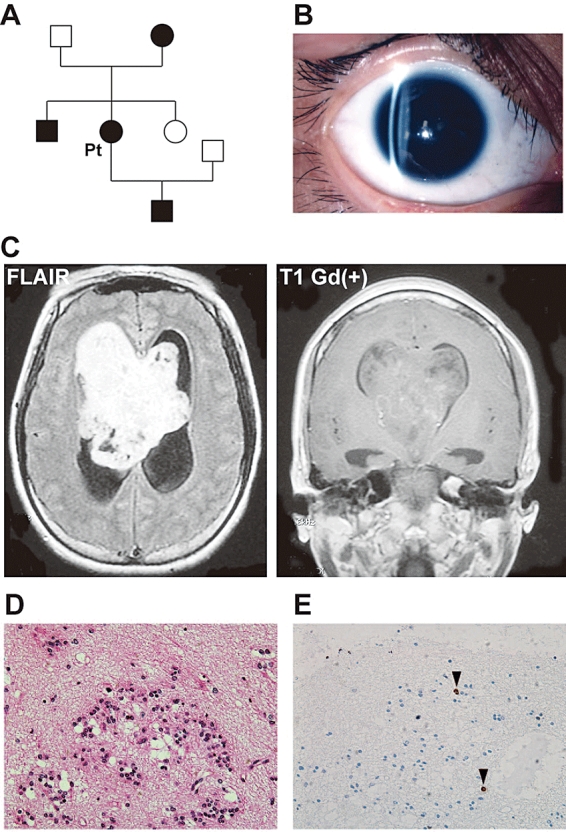
*Patient's clinical information*. **A.** Family structure of a patient with aniridia and subependymoma. Black symbols indicate individuals with aniridia. Squares represent males and circles represent females. **B.** Biomicroscopic observations of the patient with aniridia and subependymoma. **C.** Preoperative magnetic resonance imaging scans showing a tumor originating from the septum pellucidum. Left: FLAIR (fluid attenuated inversion recovery) mode, Right: T1-weighted image after administration of contrast media (Gd). The size of the tumor was 9 × 7 × 6 cm and the tumor contained multiple small calcifications and cysts. The tumor was slightly hypointense on a T1-weighted image, hyperintense on a T2-weighted image and markedly hyperintense on a FLAIR image (this image suppresses signals from cerebrospinal fluid and can obtain clear images of periventricular tumors). Tumor enhancement after administration of contrast media (Gd) was minimal. **D.** Photomicrograph of the stained tumor showing cell nests and microcysts scattered in the gliofibrillary background (stained with hematoxylin and eosin, original magnification: 200×). **E.**Photomicrograph of the tumor tissue stained with MIB-1. MIB-1-positive cells (their nuclei are colored in dark brown) are shown by arrow heads (original magnification: 200×).

The patient describes a 27-year-old female after admission to our hospital (Kanazawa University Hospital). She was born at full term after an uneventful pregnancy. Five months before admission, she complained of chronic headaches and nocturnal urinary incontinence. In March 2005, she was admitted to our hospital because of memory disturbances, unsteady gait and visual loss. Neuro-ophthalmologic examination revealed bilateral aniridia, blepharoptosis, mild cataract, papilledema, horizontal gaze nystagmus and marked and nonadjustable visual loss. Similar ocular disturbances were observed in her mother and her elder brother, but their irises were only partially defective and irregularly shaped. The maternal grandfather and three of five maternal siblings were said to have ocular disturbances but the details are unknown (Figure 1A and B). Their abnormalities of the eye had probably been overlooked because they live in a rural part of Japan and had no previous need for a specialist medical examination. Magnetic resonance imaging (MRI) on admission revealed a large tumor (9 × 7 × 6 cm) located in the third to bilateral lateral ventricles (Fig. 1C). Scattered microcalcifications were detected on computed tomography (CT). She underwent surgery via the anterior transcallosal approach. The tumor stemmed from the septum pellucidum and was well demarcated from the ventricular wall except for the right anterior horn. The tumor was grayish, rubbery, unsuckable in consistency and bled minimally. Intraoperative pathologic diagnosis indicated a subependymoma and gross total resection was performed with an Ultrasonic Surgical Aspirator (Sonopet, Miwatec Co., Ltd, Aichi, Japan).

### Histological examination

Archival paraffin sections were stained with hematoxylin–eosin (HE) and the monoclonal antibody MIB-1 (DakoCytomation, Glostrup, Denmark). The histological type and grade of tumors were re-evaluated according to the World Health Organization classification system.

### Direct sequencing

Genomic DNA was isolated from paraffin-embedded tumor tissue using RecoverAll™ Total Nucleic Acid Isolation Kit (Applied Biosystems, Foster City, CA) that contains protease to break down cross-linked proteins, and peripheral lymphocytes from the patient and from five control subjects (one male and five females) by standard methods. All the exons and exon/intron boundaries of *PAX6*, *PTEN*, *TP53* (gene for p53) and *SOX2* were screened for polymorphisms by direct sequencing of polymerase chain reaction (PCR) products. PCR was performed under the conditions described in the Supplementary Information. Direct sequencing of PCR products was performed using the BigDye Terminator v3.1 Cycle Sequencing kit (Applied Biosystems) and the ABI PRISM 3730xl Genetic Analyzer (Applied Biosystems). Polymorphisms were detected with the SEQUENCHER program (Gene Codes Corporation, Ann Arbor, MI). The information on primers, enzymes and PCR conditions used for amplification is described in Tables S1 and S2.

### Genomic quantitative PCR

All of the insertions/deletions within each gene and in intergenic regions were analyzed by real-time genomic quantitative PCR using the TaqMan method (Applied Biosystems). The *MLC1* gene at chromosome 22q13.33 was used as a normal copy number control gene. For quality control, *PFKFB1* on chromosome Xp11.21 was used to see whether our genomic quantitative PCR could accurately detect differential dosage of the X chromosome between male and female control samples. No copy number polymorphisms have been documented within these genes in the Japanese population. For the genomic quantitative PCR, DNA solutions were first quantified by an ultraviolet spectrophotometer and further quantified by a TaqMan RNase P Detection Reagent kit (Applied Biosystems). Sequences of primers for individual gene regions are listed in Table S2. Detailed information including PCR conditions is available upon request.

### Reverse transcription PCR of *PAX6* transcript from tumor tissue

Total RNA was extracted from the tumor tissue sample using RecoverAll™ Total Nucleic Acid Isolation Kit (Ambion, Austin, TX, USA). Single stranded cDNA was synthesized using High Capacity RNA-to-cDNA Master Mix (Applied Biosystems). Reverse transcription PCR (RT–PCR) amplification was performed in a nested manner: the first PCR was done using the primers set at exons 4 and 7. The second PCR was done using the primers placed on exons 5 and 7. The sequences of the PCR products were verified by direct sequencing. The detailed information of primer sequences and PCR conditions is available upon request.

### Analysis of loss of heterozygosity (LOH)

**Figure 4:**
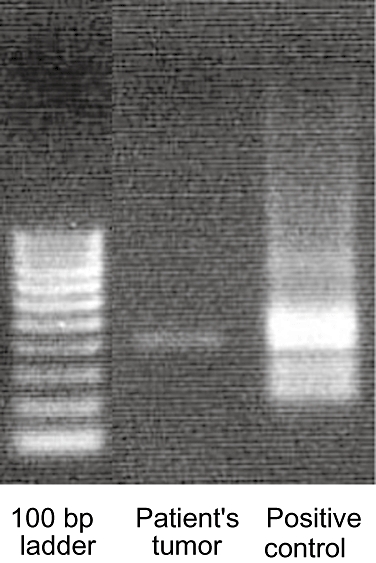
*RT–PCR analysis of PAX6 transcript from tumor tissue*. Total RNA was extracted from tumor tissue and converted to cDNA. The first PCR was done using primers designed to exon 4 and exon 7. The second (nested) PCR was done using primers set at exon 5 and exon 7. Left lane: 100 bp ladder; middle lane: nested PCR product; right lane: a positive control (human brain Marathon cDNA from BD Biosciences, San Jose, CA). The sequence of PCR product was verified by sequencing. Abbreviations: RT–PCR = reverse transcription PCR; PCR = polymerase chain reaction; *PAX6* = paired box 6 gene.

To examine the LOH, we genotyped by direct sequencing the 73 single nucleotide polymorphisms (SNPs) shown in Figure 4 and Table S7, using DNA from the patient tumor tissue, lymphocytes from the patient and DNA from the six control subjects. Detailed information including PCR primers and conditions is available upon request.

### Cytogenetic examination

Peripheral lymphocytes were cultured and chromosomes were G-banded using trypsin Giemsa (GTG) staining. Karyotyping was performed on 30 metaphase spreads per subject (21).

## RESULTS

### Histological examination of subependymoma

Histologic diagnosis indicated pure subependymoma with no other glioma component, mitosis or cellular atypism (Figure 1D and E). The MIB-1 index was less than 1%. The MIB-1 index shows the percentage of proliferating cells, in which proliferative activity was assessed by use of the monoclonal antibody MIB-1

### Sequencing and genomic quantitative PCR analyses of *PAX6*

To examine mutations of the *PAX6* gene, we performed direct sequencing of all the exons and exon/intron boundaries of the gene using DNA from the patient's tumor tissue and lymphocytes. We detected no mutations of *PAX6* in either sample.

**Figure 2:**
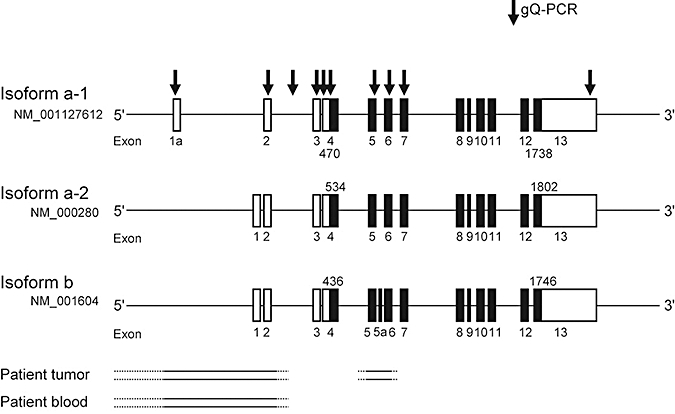
*Genomic structures of the three PAX6 isoforms*. In isoform a-1, A of the ATG codon is located at nucleotide (nt) position 470 and the end of the stop codon at nt 1738, counted from the 5′-end of the first exon. In isoform a-2, A is located at nt 534 and the end of the stop codon at nt 1802. In isoform b, A is located at nt 436 and the end of the stop codon at nt 1746. The positions where we performed gQ–PCR are shown in red. The genomic structure of *PAX6* is based on the University of California Santa Cruz (UCSC) March 2006 draft assembly of the human genome database (http://genome.ucsc.edu/cgi-bin/hgGateway?org=human). Information on known single nucleotide polymorphisms was obtained from the National Center for Biotechnology Information (NCBI) database (http://www.ncbi.nlm.nih.gov/). At the bottom, deleted portions are shown. Solid lines show experimentally verified regions. Dashed lines show that the breakpoints are located somewhere in the regions. Abbreviations: *PAX6* = paired box 6 gene; gQ–PCR = genomic quantitative polymerase chain reaction.

**Figure 3:**
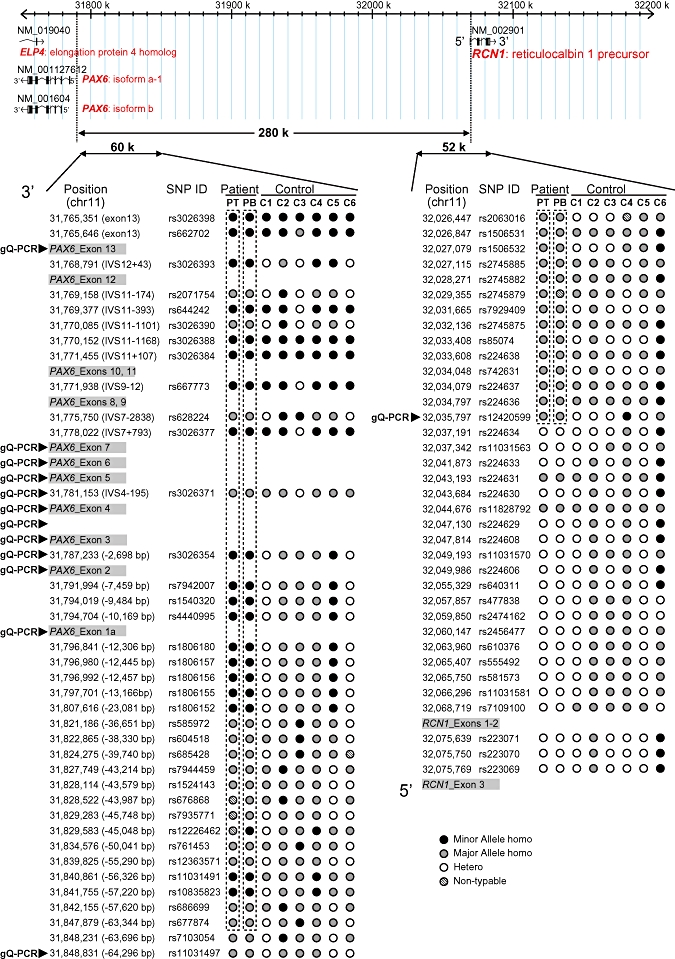
*Analysis of loss of heterozygousity around PAX6 on chromosome 11p from the patient's tumor tissue and lympocytes and six control samples*. Stretches of homozygous SNPs are encircled by dotted lines. Exons are shaded. Minor allele is defined as a frequency of <5%. The positions of SNPs located at the 5′-upsteam region of *PAX6* are shown as distances from the A of the ATG codon located in exon 4 of the gene (also see Figure S1). Abbreviations: PT = patient's tumor tissue; PB = patient's lymphocytes; C = control; gQ–PCR = genomic quantitative polymerase chain reaction; *PAX6* = paired box 6 gene; SNPs = single nucleotide polymorphisms.

Next, to examine allelic loss of *PAX6* in the patient's tissue, we performed genomic quantitative PCR with probes designed to exons 13, 7, 6, 5, 2 and 1a of *PAX6* (Figure 2), and around rs11031497 [64 296 base pairs (bp) upstream from the A of the ATG codon of *PAX6* and 235 016 bp downstream of the 3′-end of the last exon of *RCN1*] and rs12420599 (251 262 bp upstream from the ATG codon of *PAX6* and 48 050 bp downstream of the 3′-end of *RCN1*) (Figure 3). In the lymphocyte genome, hemizygous deletions were evident with probes to exons 2 and 1a, indicating that the deleted region spans from between exons 5 and 2 to downstream of rs11031497 (Table 1). In the tumor tissue, the probe to exon 7 showed no evidence of deletion and the probe to exon 6 displayed a signature of deletion (Table 1). Therefore, it seemed that in the tumor cells, the *PAX6* deletion extends more to 3′-direction than that of the lymphocyte genome, or the tumor genome has an additional de novo deletion. To address this issue, we performed additional genomic quantitative PCR analyses with probes designed to intron 2, exon 3, intron 3, exon 4 and intron 4 (Figure 2). These results show that the tumor genome has two distinct *PAX6* deletions (Table 1): the one is the same to that of normal somatic cells (albeit we did not examine whether the 5′-break points of tumor and somatic cell genomes are on the same position), and the other de novo one spans from the intron 4 to intron 6 of *PAX6* gene (Figure 2). We also attempted to determine the deletion boundaries of the de novo deletion by PCR, but we failed to amplify any genomic fragments under multiple conditions tested, presumably because it is difficult to completely eliminate peptide fragments bound to DNA, which is generated by formaldehyde fixation of tumor tissue; we cannot amplify such DNA of relatively long size by PCR that peptide fragments are attached to.

**Table 1:** Genomic quantitative–PCR of *PAX6*. Abbreviations: PCR = polymerase chain reaction; *PAX6* = paired box 6 gene.

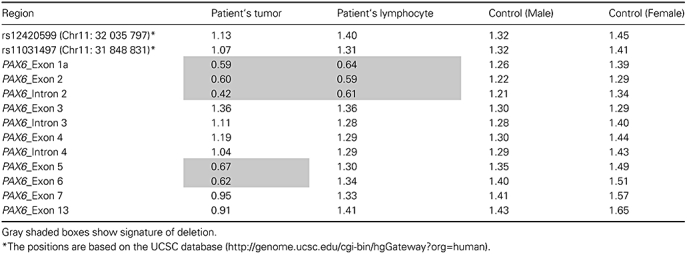

### RT–PCR analysis of *PAX6* transcript from tumor tissue

Next, we set out to determine whether the two different deletions of *PAX6* in tumor genome are on the same chromosome or on different chromosomes. The 5′-side (germline) deletion involves the promoter region and the first two exons, making the mRNA transcription impossible (Figure 2). Therefore, if the two different deletions were located on the same chromosome, we could not amplify any portions of *PAX6* transcript from the tumor tissue including the region that corresponds to the 3′-side de novo deleted genomic interval. Our RT–PCR analysis detected the cDNA stretch that spans from exon 5 to exon 7 (Figure 4). These results suggest that the two deletions seen in the tumor genome are likely to be located on the same chromosome in tandem. However, we cannot completely exclude the possibility that the detected PCR products stemmed from the contaminated normal cells in the tumor tissue.

### LOH analysis around *PAX6* locus

We examined the genotypes of 73 SNPs mapped in the interval spanning from the last exon 13 of *PAX6* to the 5′-region of the neighboring *RCN1* gene (Figure 3). We included all known SNPs that mapped in the region from intron 2 to the exon 13, to confirm the genomic quantitative PCR results for the position of the breakpoint in tumor cells. In both the tumor and lymphocyte samples, the homozygosity of SNPs ensued from the most 3′-end SNP examined, e.g. rs3026398–rs12420599. The 5′-neighboring SNP to rs12420599, rs224634 showed heterozygosity in both samples. In some of the five control samples (lymphocytes), heterozygous genotypes of SNPs were detected in the described genomic stretch (Figure 3). These results suggest that (i) the 3′-end of the deleted region from the patient lymphocytes is within intron 2 of *PAX6* as deduced from the combined genomic quantitative PCR and LOH data, and (ii) the 5′-ends of the deleted regions are the same or very close to each other in the tumor and normal somatic cells of the patient. In addition, the results indicate that the genomic deletions occurring in the patient do not involve the *RCN1* gene or are unlikely to involve the promoter region of the *RCN1* (the distance between rs224634 and the 5′-end of *RCN1* is 33 256 bp). From LOH analysis, we could not obtain further informative data beyond quantitative genomic PCR analysis, regarding the 3′-ends of the two deletions, because there were no heterozygous SNPs in the interval 3′-downstream of intron 2 [and there are no known SNPs in the genomic stretch from exon 5 to exon 7 (Figure 3)].

### Screening of the tumor related genes: *PTEN*, *TP53* and *SOX2*

To further evaluate the potential genomic abnormalities associated with this subependymoma, we examined three tumor-related genes: *PTEN*, *TP53* and *SOX2*. We performed direct sequencing of all exons and exon/intron boundaries of the three genes from patient tumor tissue and lymphocytes and lymphocytes from six control subjects. In this analysis, we detected two novel SNPs (one in intron 1 and the second in intron 8) within *PTEN* (Table S3). A novel C allele at IVS4-137 was found in the patient's tumor. The other novel 8T allele at IVS4-30-37 (insT: 9T is already registered) was found in the patient's tumor. We deposited these novel SNPs into the NCBI database, and obtained the I.D. ss161110057 and ss161110058, respectively. However, there were no exonic mutations in any of the samples. In addition, the genotypes of the examined SNPs located between the first and last intron of *PTEN* were all heterozygous in the tumor sample (Table S3), excluding the possibility of allelic deletion within the gene.

With regards to the *TP53* gene, sequencing detected no mutations in either the patient's tumor or lymphocyte samples, or in the two control samples. The genotypes of all the SNPs examined in the patient were homozygous in the tumor tissue and lymphocytes, giving no information about the genomic deletions (Table S4). We confirmed the absence of any deletions using real-time PCR to quantify genomic DNA templates from exon 1, intron 6 and exon 11 (the last exon) (Table S5).

The *SOX2* gene consists of one exon. We performed genomic quantitative PCR using probes designed to the 5′and the 3′-regions of exon1. The results showed no evidence of genomic deletions (Table S6).

### Cytogenetic analysis and genomic quantitative PCR analyses of chromosomes 6q and 14q

It was reported that there were trisomy of chromosome 7, monosomy of chromosome 8 and partial losses on chromosome 6q and chromosome 14q in some subependymoma cases (10) (Figure S3). Therefore we examined chromosomal aberrations. Karyotype analysis of lymphocytes did not detect any chromosomal rearrangements, aneuploidies and gross deletions and duplications (Figure S1). In addition, we performed genomic quantitative PCR placing probes at reported regions chromosome 6q and chromosome 14q (9) (Table 2). The results showed no evidence of genomic deletion in chromosomal 14q, but showed that there is a region of hemizygous loss in chromosome 6q (Table 2).

**Table 2:** Genomic quantitative RT–PCR analyses of chromosomes 6q and 14q. Abbreviation: RT–PCR = reverse transcription polymerase chain reaction; Chr = chromosome.

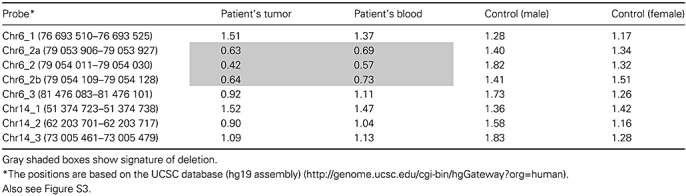

## DISCUSSION

Here, we report the first case of subependymoma developed in a patient with a hereditary disease (aniridia). The size of subependymoma was unusually huge. We also examined the underlying mechanisms and detected a deletion in the 5′-region of *PAX6* in lymphocytes and tumor cells and an additional de novo deletion in the tumor cells from the patient. Postoperatively, the patient was slightly hemiparetic on the left, but recovered completely after 1 month. A ventriculoperitoneal shunt procedure was performed for delayed hydrocephalus. Three years after the operation, an MRI revealed no tumor recurrence and she recovered fully from her neurological symptoms. During this time, she had gotten married and delivered a full-term baby boy who also displayed an irregular-shaped iris and spontaneous nystagmus (Figure 1A).

While the familial aniridia can be explained by the *PAX6* deletion, it is likely other/additional genetic mechanism(s) played a role in the onset and extraordinarily large growth of the subependymoma. Therefore, we examined three other tumor suppressor genes, *PTEN*, *TP53* and *SOX2*, which are relevant to either glioma or PAX6 function. *PTEN* is a major tumor suppressor gene that is inactivated in 50% of high-grade gliomas (5). *PAX6* and *PTEN* expression levels are deemed to be two independent and powerful prognostic markers for the outcome of patients with astrocytic malignant gliomas (24). The mutational inactivation of *TP53* has been reported in progressive glioblastomas (5). *SOX2* encodes a transcription factor, and this protein is known to act by forming a heterodimer with PAX6 (9). SOX2 is a marker for gliomas in early and progressed stages, and it plays a fundamental role in the maintenance of the self-renewal capacity of neural stem cells after they have acquired cancer properties (6). However, the patient had no mutations or deletions in these genes ruling out a possible contribution to the development of the giant subependymoma. In addition, karyotype analysis showed that there are no gross chromosomal abnormalities in the patient. Regarding microdeletions reported in some subependymoma cases (10), we detected no deletion in chromosome 14q, but found a partial loss in chromosome 6q (Table 2, Figure S2). However, since the latter genomic portion is one of the known common copy number polymorphisms (13), it is likely that there are little effects of the deletion on the tumor onset and growth.

The gene immediately upstream of *PAX6* is the gene (*RCN1*) for reticulocalbin 1 EF-hand calcium-binding domain (*PAX6* and *RCN1* are located in a head-to-head fashion in the genome). There are some reports that *RCN1* is associated with tumors (3). However, the detected deletion in the tumor cells did not affect this gene, again excluding a potential role for *RCN1* in this subependymoma.

There are several reports demonstrating that deletions of tumor-related regions of *PAX6* or dominant negative activity generated by *PAX6* mutations are associated with tumors of glial cell origin. A 57 bp *cis*-regulatory element named E1E (exon 1 enhancer) in the first exon of *PAX6* plays an important role in the expression of this gene in glioblastoma cell lines (23), and a powerful silencer (SX250) located between 1518 and 1268 bp from the A of the ATG codon is able to repress the promoter activity of *PAX6* in cervical carcinoma and glioblastoma cell lines (22). Pure subependymomas have a dense gliofibrillary background (18). In the patient's subependymoma cells, the above regulatory elements are heterozygously deleted. Therefore, it would be one of possible scenarios that the subependymoma occurred independently in this patient but its unusually large size (maximum diameter of 90 mm) is due to haploinsufficiency of tumor suppressor elements. It is known that PAX6 proteins with C-terminal deletions have dominant negative activities (19). In this study, we detected an additional de novo *PAX6* 3’-side deletion in patient's tumor cells. Our PCR analysis of transcripts suggested that the tumor cell has the two distinct deletions on the same chromosome, although we cannot completely exclude the possibility that the results are derived from the coexisting normal cells in the tumor tissue. If both deletions were located on the same chromosome, the effects of PAX6 dominant negative activity could be neglected because the gene is transcribed from only the normal allele.

The incidence of all types of intracranial neoplasms is reported to be 0.008%–0.01% in general population (11). And the incidence of subependymoma is reported to be 0.5% of all intracranial neoplasms (15). Therefore the incidence of subependymoma is deemed to be 0.004%––0.005% in both general population and aniridia patients. We think that this rareness is related to no reports, to our knowledge, of giant subependymomas in aniridia patients. In addition, aniridia itself is very rare: a frequency of about 1 in 50 000–100 000 live births (2).

It is reported that a compound heterozygous mutation of *PAX6* causes severe defects of the brain including the eyes (7). However, we did not detect any amino acid mutations of PAX6 in the patient's tumor or lymphocytes. For epigenetic changes, it is known that there are many CpG-rich regions in *PAX6*(17). These regions may be frequently methylated in tumors, which could affect the transcription of *PAX6* and tumorigenesis, though we did not address this issue in the present study because we do not have neighboring normal tissue, making the comparison of DNA methylation status between tumor and normal tissues impossible.

It is noteworthy that although *PAX6* defects elicit congenital aniridia in a dominant hereditary fashion, phenotypes of the eye are sometimes overlooked, especially when the abnormalities are mild and/or de novo gene defects occur. Our experience suggests that it would be prudent to pay closer attention to abnormalities of the eye when presented with large, indolent, benign gliomas.

In summary, we identified the first case of subependymoma concomitant with a hereditary disease, familial aniridia. In addition, the present subependymoma is uncommon in terms of its extremely large size, anatomical site of development and the patient's age and sex. There is a unique registration site, the Danish Cancer Registry, that has collected data on patients having both aniridia and cancer since 1943 (20). Although eight patients are diagnosed as having kidney, lung and digestive tract cancers from the 136 aniridia carriers, there are no recorded brain tumors (8). Therefore, the currently detected deletions of *PAX6* are unlikely to be responsible for the subependymoma onset. Given the reports that loss of *PAX6* function leads to aberrant proliferation and immature differentiation of astrocytes in rodents (16), it is reasonable to assume that the *PAX6* deletions contributed to the unusual proliferation of the tumor after its independent initiation. Further reports of the mechanisms of development of subependymoma are warranted.

## SUPPORTING INFORMATION

**Figure S1.***GTG-banded photographs of patient's chromosomes*. Note that there are no karyotypic abnormalities in the patient's lymphocytes.

**Figure S2.***Chromosomes 6q and 14q that were examined for deletions*. Horizontal arrows show the positions of the probes used for genomic quantitative PCR analyses (see Table 2 in the main text). The positions (bp) of the BACs (bacterial artificial clones), RP11-398K22, RP-202D23, RP-332O9 and RP11-203D6 (1), are also shown according to the UCSC database (hg19 assembly) (http://genome.ucsc.edu/cgi-bin/hgGateway?org=human). On each chromosomal region, the hemizygous deletion of the interval between the denoted BACs is reported in some subependymoma cases (1). For red symbols at the left of horizontal arrows, see Table 2.

**Table S1.** Primer sequences and PCR conditions used for *PAX6*, *PTEN*, *TP53* and *SOX2* screening.

**Table S2.** Primer and probe sequences used for gQ-PCR.

**Table S3.** SNP genotypes of *PTEN*.

**Table S4.** SNP genotypes of Homo sapiens tumor protein *p53 (TP53)* gene.

**Table S5.** Genomic quantitative-PCR of *TP53*.

**Table S6.** Genomic quantitative-PCR of *SOX2*.

**Table S7.** LOH examination of *PAX6*.
