# Supplementary figures and images for: Giant Subependymoma Developed in a Patient with Aniridia: Analyses of PAX6 and Tumor-relevant Genes
Source: Brain Pathol. 2010 Nov;20(6):1033–41. doi: 10.1111/j.1750-3639.2010.00406.x (PMC2991767; doi:10.1111/j.1750-3639.2010.00406.x)

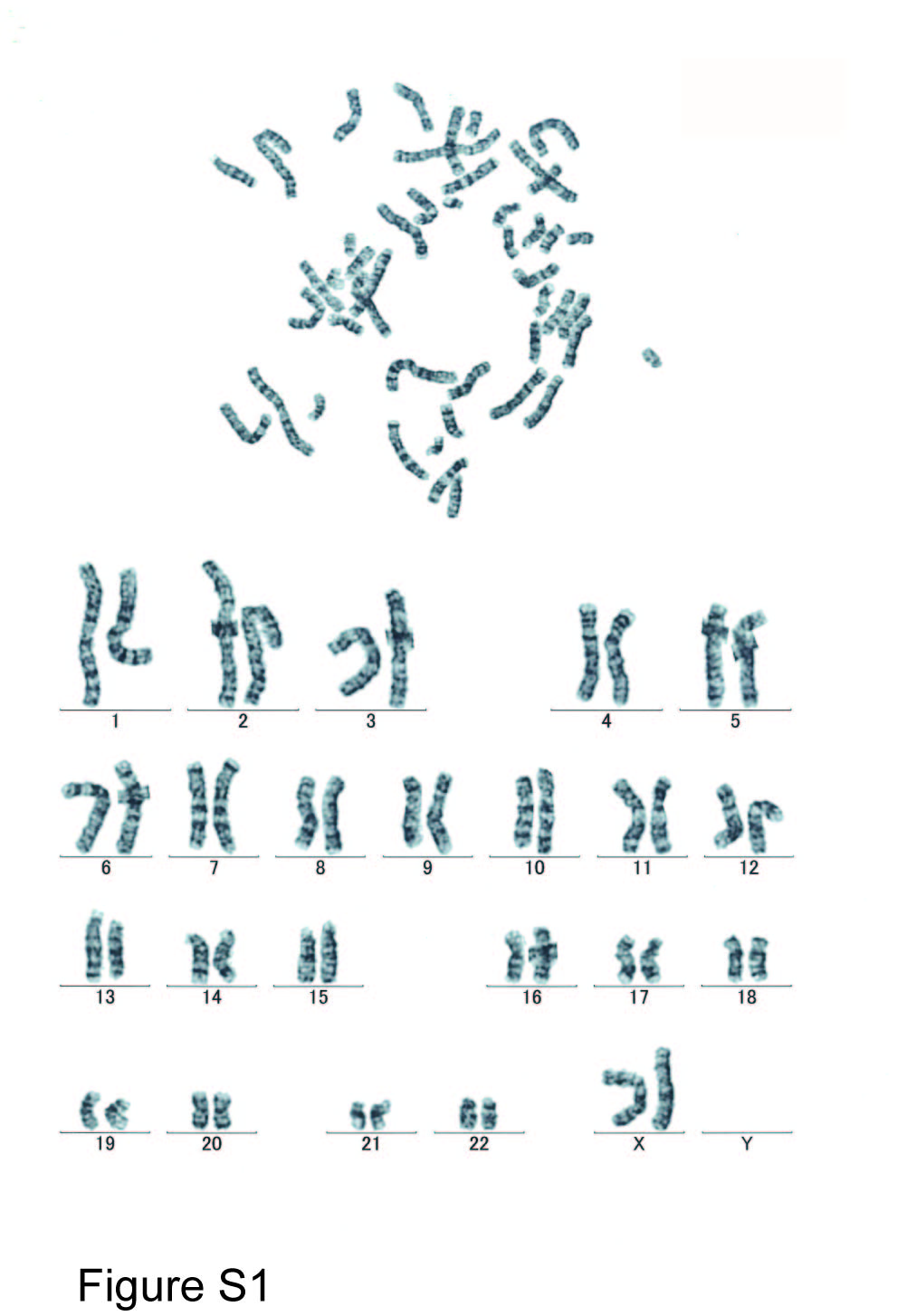

Supplement: Supplementary file 1 [file bpa0020-1033-SD1.jpg]

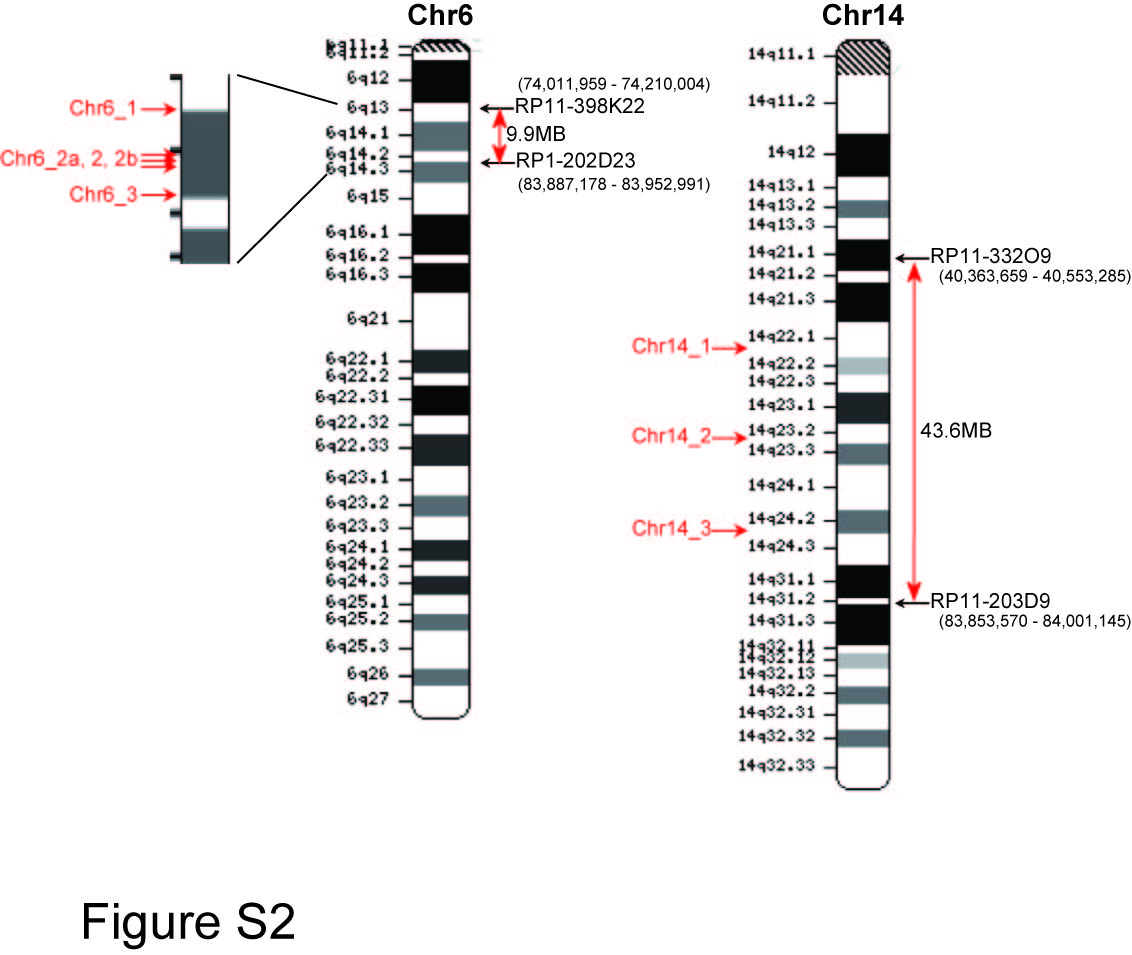

Supplement: Supplementary file 2 [file bpa0020-1033-SD2.jpg]
